# Novel Heterogenous *CHS1* Mutations Identified in Five Japanese Patients with Chediak-Higashi Syndrome

**DOI:** 10.1155/2010/464671

**Published:** 2010-12-15

**Authors:** Fuminori Tanabe, Hirotake Kasai, Michiko Morimoto, Shigeharu Oh, Hidetoshi Takada, Toshiro Hara, Masahiko Ito

**Affiliations:** ^1^Department of Human Science, Interdisciplinary Graduate School of Medicine and Engineering, Faculty of Medicine, University of Yamanashi, 1110 Shimokato, Chuo, Yamanashi 409-3898, Japan; ^2^Department of Microbiology, Interdisciplinary Graduate School of Medicine and Engineering, Faculty of Medicine, University of Yamanashi, 1110 Shimokato, Chuo, Yamanashi 409-3898, Japan; ^3^College of Nursing Art and Science, University of Hyogo, Akashi, Hyogo 673-8588, Japan; ^4^Shizuoka Children's Hospital, Shizuoka 420-8660, Japan; ^5^Department of Pediatrics, Graduate School of Medical Science, Fukuoka 812-8582, Japan

## Abstract

Chediak-Higashi syndrome (CHS) is a rare, autosomal recessive disorder characterized by oculocutaneous albinism, recurrent bacterial infections and progressive neurological dysfunction. We demonstrate novel heterogenous mutations of *CHS1*, the responsive gene of CHS, identified in five Japanese patients with CHS. Patients 1, 2, and 3 were siblings, and they had albinism of the skin and hair. They all had a heterogenous two-base deletion (c.5541-5542 del AA, p.Q1847fsX1850) in exon 18. Patient 4 had a heterogenous single-base insertion (c.3944-3945 ins C, p.T1315fsX1331) in exon 10. The patient exhibited severe early-onset phenotype and suffered from hemophagocytic lymphohistiocytosis. Patient 5 had two heterogenous nonsense mutations; c.7982C>G, p.S2661X in exon 30 and c.8281A>T, p.R2761X in exon 31. The patient suffered from infections in childhood and had visual disturbance and albinism of the skin and hair. The *CHS1* mutations described here have not been reported previously.

## 1. Introduction

Chediak-Higashi syndrome (CHS; MIM 214500) is an autosomal recessive disorder characterized by oculocutaneous albinism, increased susceptibility to pyogenic infections, defective natural killer (NK) activity, delayed bactericidal activity of neutrophils, and the presence of giant lysosomes in many cell types [1–3]. We previously reported that abnormally downregulated protein kinase C activity is responsible for the impaired cellular functions of polymorphonuclear leukocytes, fibroblasts and NK cells of CHS mice and patients [4–9]. The manifestation of CHS may result from defective trafficking of proteins into late multivesicular endosomes [10]. Most CHS patients die young due to a lymphoproliferative histiocytosis called the accelerated phase unless they undergo bone marrow transplantation.

 The genetic defect resulting in CHS was identified in 1996 [11, 12]. The human gene, *CHS1*, was also called *LYST* (Lysosomal Trafficking Regulator). A similar disorder has been identified in beige mice and many other mammalian species. Human CHS patients and beige mice have homologous disorders associated with the *CHS1 *mutation [11–13]. *CHS1* consists of 51 coding exons with an open reading frame of 11,406 bp [12]. The CHS1 protein is cytosolic and is composed of 3801 amino acids with a molecular weight of 429 kDa. It is known that CHS1 has a pleckstrin homology domain, a BEACH domain, and WD-40 repeats in the C-terminal region [14]. While the exact function of the CHS1 protein has not been elucidated, the protein suggested to regulate lysosomal size or lysosomal fission and affect cellular events, such as those of nuclear phosphatidylinositol-4, 5-bisphosphate [15, 16].

 Thus far, 31 mutations in the *CHS1* gene have been reported, including frameshift, nonsense, and missense mutations [17–21]. Only five Japanese CHS patients have been examined to date. Two patients had a one-base substitution, and one patient had a deletion, whereas no mutation of the *CHS1* gene was detected in the other two patients [17]. Thus, we attempted to examine the mutations in other Japanese CHS patients. Here, we report novel heterogenous mutations of the *CHS1 *gene identified in Japanese patients with CHS.

## 2. Patients and Methods

Informed consent for this study was obtained from the patients or their parents. The study protocol was approved by the Ethics Committee of the University of Yamanashi.

**Figure 1:**
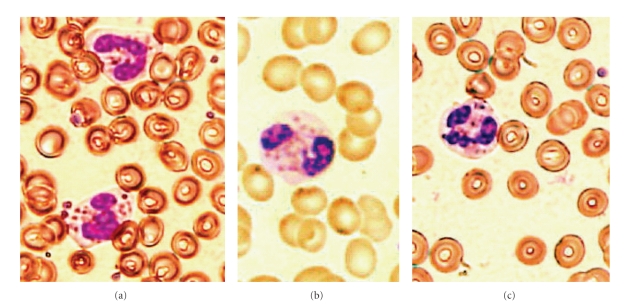
Wright stain of peripheral blood smear of CHS patients, showing polymorphonuclear leukocytes with giant granules (200×). (a) Patient 1, (b) patient 2, and (c) patient 3.

**Figure 2:**
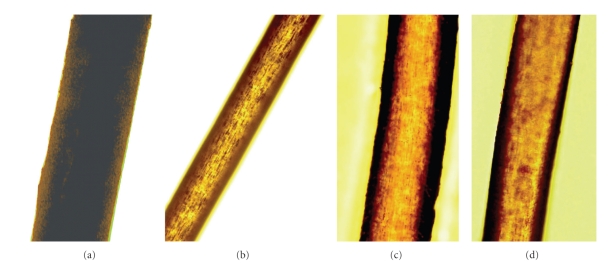
Hair shafts under light microscopy (200×). (a) Hair sample of normal Japanese control, showing evenly distributed pigment. (b) Patient 1, (c) Patient 2, (d) Patient 3. Hair shafts of CHS patients showing atypical granular pigment pattern.

Patients 1, 2, and 3 (siblings) were 23-year-old male, 20-year-old female, and 17-year-old female, respectively. Giant granules were observed in polymorphonuclear cells from these three patients (Figure 1). They all had albinism of the skin and hair (Figure 2). However, there was no history of severe infection in any patient. Their parents were normal and healthy. 

**Figure 3:**
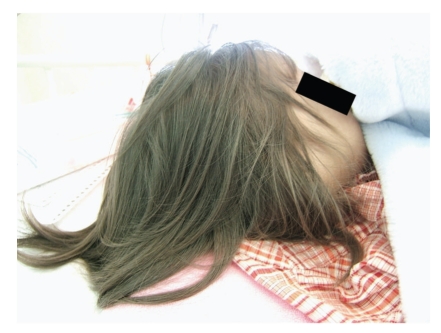
Hair and skin of patient 4 at the age of 4, showing silvery hair and hypopigmentaion of skin.

**Figure 4:**
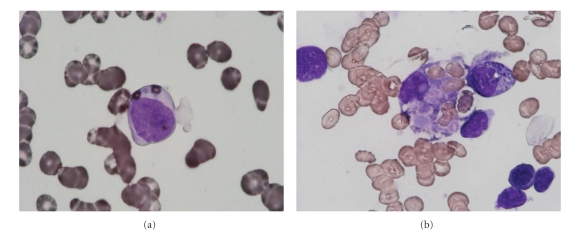
Bone marrow aspirate smear of patient 4, showing (a) myeloperoxidase-positive giant granules and (b) hemophagocytosis.

Patient 4 was a 6-year-old female with visual disturbance and hypopigmentation of the skin and hair (Figure 3). The diagnosis of CHS was determined by the presence of myeloperoxidase-positive giant granules in leukocytes (Figure 4(a)). At the age of 4, she suffered from hemophagocytic lymphohistiocytosis (Figure 4(b)), which is known as accelerated phase. At the time of the study, she had high fever, bleeding tendency, hepatosplenomegaly, and pancytopeny. She had low NK activity and decreased bactericidal activity of neutrophils. In addition, she had been treated with cyclosporine and steroids and then underwent bone marrow transplantation.

Patient 5 was a 21-year-old female who suffered from recurrent infections in childhood. At the time of the study, she had visual disturbance and neurological dysfunction including gait disturbance. Detailed information on this patient was not available.

Peripheral blood samples were obtained from five CHS patients who had not undergone bone marrow transplantation. Blood samples from parents of patients 1, 2, and 3 were also obtained. Genomic DNA was obtained from blood samples. Extraction of DNA was performed using the QIAamp DNA Blood kit (QIAGEN Inc., Valencia, CA, USA) according to the manufacturer's protocols. Polymerase chain reaction (PCR) primer pairs (25–35 bp) designed from intron sequences flanking each exon were used to amplify genomic DNA segments spanning each exon. For example, exons 9-10 were amplified using the F primer 5′-ATTTTTGCCACTAGATCTTCTAAATG-3′, and R primer was 5′-AGAAGCCATTATTATCAACTTTTCAC-3′. The F primer of exon 18 was 5′-TGCTACTGGCCACTAAGGTTGTGTGTC-3′, and R primer was 5′- GACTTTGATGACGAGATGAGTATCACTGC-3′. The F primer of exon 30 was 5′-CATTGTATCTATTACATCTAATACACCTGATACAC-3′, and R primer was 5′-ACGTATAATACAGTCAACATAAAACCTCTATTTCC-3′. PCR was performed using KOD-Plus-Ver. 2 according to the manufacturer's protocols (TOYOBO, Tokyo, Japan). The PCR products (220–3600 bp) were separated by electrophoresis on agarose gels. DNA was isolated from each band using the QIAquick Gel Extraction kit (QIAGEN Inc.). Direct sequencing using dye termination cycle sequencing was performed at FASMAC Co., Ltd. (Atsugi City, Kanagawa, Japan). The mutations were analyzed using Sequence Scanner Ver. 1 (Applied Biosystems, Tokyo, Japan) and were also checked at the cDNA level. For this purpose, total RNA was extracted from blood samples using Isogen-LS (Nippon Gene Co., Ltd., Tokyo, Japan) according to the manufacturer's protocols. cDNA was synthesized using PrimeScript reverse transcriptase (Takara Bio. Inc., Shiga, Japan), and PCR was performed as described above.

## 3. Molecular Analysis

**Figure 5:**
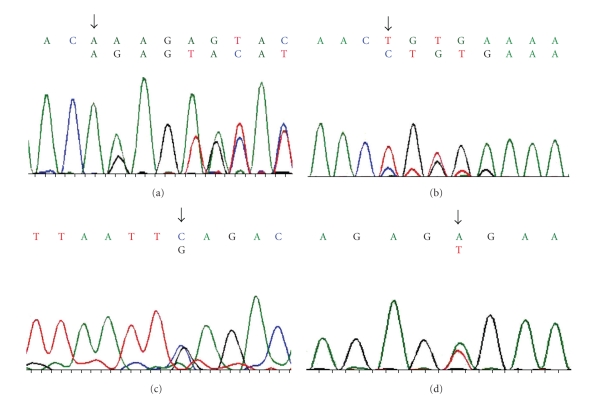
The cDNA sequence patterns of *CHS1* mutations. (a) Patients 1, 2, and 3: frameshift mutation in exon 18, c.5541-5542delAA, p. Q1847fsX1850. These three patients were siblings. The same sequence pattern of cDNA was observed in their father. (b) patient 4: Frameshift mutation in exon 10, c.3944-3945 ins C, p.T1315fsX1331. (c) patient 5: Nonsense mutation in exon 30, c.7982 C > G, p.S2661X. (d) patient 5: Nonesense mutation in exon 31, c.8281 A > T, p.R2761X.

All 51 exons of the *CHS1* gene of the five patients from three families were sequenced, and four patterns of novel heterogenous mutations were identified. In patients 1, 2, and 3, a two-base deletion (c.5541-5542 del AA) in exon 18 resulted in a frameshift mutation that eventually led to the formation of a stop codon (p.Q1847fsX1850) (Figure 5(a)). The second mutation was not found in the coding exons. The same heterogenous mutation was detected in their father. In their mother, no mutation was found in the *CHS1* exons. Since their parents had no symptoms, it was possible that the second mutation lies in the intron sequence or splice mutation site. It was also possible that this mutation shows a mild phenotype associated with heterozygosity. Another possibility is the mutation in another gene, which affects the generation of lysosome-related organelles.

In patient 4, we detected a heterogenous one-base (C) insertion (c.3944-3945 ins C) in exon 10, resulting in a frameshift mutation that led to the formation of a stop codon (p.T1315fsX1331) (Figure 5(b)). The second mutation was not found in any coding exon. The blood samples of her parents were not available. 

In patient 5, two heterogenous mutations were identified; C-G substitution (c.7982 C > G) in exon 30 resulted in a nonsense mutation (p.S2661X) (Figure 5(c)), and A-T substitution (c.8281A > T) in exon 31 resulted in a nonsense mutation (p.R2761X) (Figure 5(d)). Blood samples of her parents were not available.

## 4. Discussion

The sequence pattern of *CHS1* mutations described here has not been reported previously. All mutations were predicted to halt production of the complete CHS1 protein. Karim et al. [17] demonstrated that missense mutant alleles that likely encode CHS1 polypeptide with partial function were found in adolescent and adult forms of CHS, whereas functionally null mutant *CHS1 *alleles were found in case with severe childhood CHS. Westbroek et al. [19] also demonstrated that cellular defects in CHS correlate with the molecular genotype and clinical phenotype. In the present study, patient 4 and 5 exhibited early-onset CHS and were predicted to have a truncated CHS1 protein. Although the other three patients (patients 1, 2, and 3) were also predicted to have the truncated protein, they had clinically milder forms of CHS. In these patients, only single mutation in the *CHS1* coding exons was detected. Since these patients were relatively healthy, it is possible that the mutant protein is acting as a dominant negative, resulting in a mild phenotype. In addition, we cannot exclude a possibility that the second mutation lies in the intron sequence or splice mutation site. However, the real reasons remain unknown, as we could not examine protein levels in these patients. In the previous report [17], no mutation in the *CHS1 *gene was found in 10 CHS patients, and only single mutations were found in 4 CHS patients. These findings suggest a possibility that the mutation lies in a gene other than *CHS1*, which affects the generation of lysosome-related organelles. In patient 5, two heterogenous nonsense mutations were identified. Recently, two heterogenous nonesense mutations in the *CHS1* gene were reported in an African-American patient [20]. In that report, the patient exhibited severe childhood CHS. These findings support that the functionally null *CHS1* mutant alleles are detected in severe childhood CHS.

In Japanese CHS patients, only three mutations have been identified so far, whereas no mutation in the *CHS1* gene was found in two other patients [17]. We have described four patterns of novel mutations that are expected to result in a truncated CHS1 protein. However, the relationship between these mutations and the phenotype remains to be resolved. Examination of a large number of CHS patients will be required to clarify the genotype-phenotype correlation.
